# Circular RNA circ_0020123 promotes non-small cell lung cancer progression by sponging miR-590-5p to regulate THBS2

**DOI:** 10.1186/s12935-020-01444-z

**Published:** 2020-08-11

**Authors:** Liang Wang, Lantao Zhao, Yonghong Wang

**Affiliations:** Department of Thoracic Surgery, Lianyungang Second People’s Hospital, No. 41 Hailian East Road, Haizhou District, Lianyungang, 222006 Jiangsu China

**Keywords:** NSCLC, Circ_0020123, miR-590-5p, THBS2

## Abstract

**Background:**

The incidence and death rate of non-small cell lung cancer (NSCLC) in China ranks the first among the malignant tumors. Circular RNA (circRNA) was reported to be involved in the progression of NSCLC. Our study aimed to investigate the underlying mechanism of circ_0020123 in NSCLC progression.

**Methods:**

Quantitative real-time polymerase chain reaction (qRT-PCR) was used to detect the expression of circ_0020123, miR-590-5p and Thrombospondin 2 (THBS2) in NSCLC tissues and cells. Cell proliferation and migration were examined by Cell Counting Kit-8 (CCK-8) assay and Transwell assay, respectively. Flow cytometry assay was used to detect the apoptosis of NSCLC cells. The protein levels of Ki-67, matrix metalloprotein-9 (MMP-9), Cleaved-caspase9 (Cleaved-casp9) and THBS2 were detected by Western blot. The targets of circ_0020123 and miR-590-5p were predicted by starBase 3.0 and TargetScan, and then confirmed by dual-luciferase reporter assay and RNA immunoprecipitation (RIP) assay. The animal experiment showed the effect of circ_0020123 on tumor growth in vivo.

**Results:**

The expression of circ_0020123 was upregulated in NSCLC tissues and cells. Functionally, circ_0020123 downregulation inhibited the proliferation and migration and promoted the apoptosis of NSCLC cells. Interestingly, circ_0020123 directly targeted miR-590-5p, and inhibition of miR-590-5p reversed the knockdown effects of circ_0020123 on NSCLC cells. More importantly, THBS2 was a target of miR-590-5p, and THBS2 overexpression reversed the effects of circ_0020123 knockdown on cell proliferation, migration and apoptosis in NSCLC cells. Finally, suppression of circ_0020123 inhibited tumor growth in vivo through miR-590-5p/THBS2 axis.

**Conclusion:**

Circular RNA circ_0020123 regulated THBS2 by sponging miR-590-5p to promote cell proliferation and migration and inhibit cell apoptosis in NSCLC cells.

## Highlights

1. Circ_0020123 was upregulated, and downregulation of circ_0020123 inhibited cell proliferation, migration and promoted cell apoptosis in NSCLC cells.
2. Circ_0020123 directly targeted miR-590-5p and miR-590-5p downregulation reversed the knockdown effects of circ_0020123 on NSCLC progression.
3. THBS2 acted as a target of miR-590-5p, and overexpression of THBS2 reversed the effects of circ_0020123 knockdown on NSCLC progression.
4. Downregulation of circ_0020123 suppressed tumor growth *in vivo* through miR-590-5p/THBS2 axis.

## Background

Lung cancer has the highest incidence (11.6% of total cases) and is the most common cause of cancer death (18.4% of total cancer deaths) in worldwide [1]. Lung cancer can be divided into several histological subtypes according to the location and the tendency of metastasis. Small cell lung cancer (SCLC) accounts for about 15% of all lung cancer cases [2]. However, non-small cell lung cancer (NSCLC) accounts for 85% of lung cancer, and the 5 years overall survival rate (OS) is only about 15% [3]. Therefore, it is important to find the effective treatment and potential molecular targets of NSCLC progression.

Circular RNA (circRNA) is a single stranded RNA molecule with a closed circular structure. Recently, amounts of circular DNA have been discovered, and most of which were thought to be the by-products of typical splicing [4, 5]. Previous reports indicated that the expression of circRNA was tissue-specific and the change of its expression intensity was associated with some diseases [6–8]. Furthermore, circRNA was involved in the occurrence and development of the disease and might be used as a potential biomarker in clinical diagnosis, prognosis and treatment of diseases [9, 10]. For example, circ_0039569 facilitated cell proliferation and migration of renal cell carcinoma by sponging miR-34a-5p to regulate CC Chemokine ligand 22 (CCL22) [11]. Meanwhile, hsa_circ_0043256 participated in the progression of NSCLC cells by mediating the cinnamaldehyde treatment [12]. A previous report suggested that circ_0020123 acted as an oncogene in NSCLC, and circ_0020123 regulated zinc-finger-enhancer binding protein 1 (ZEB1) and enhancer of zeste homolog 2 (EZH2) by competitively binding with miR-144 to induce cell progression and migration [13]. These reports suggested that circ_0020123 was a vital factor in the pathogenesis of NSCLC, and its function and molecular mechanism need to be further studied.

As a small endogenous RNA, microRNA (miRNA) is essential in regulating gene expression and plays a potential role in the exploitation of biomarkers [14]. Recently, some aggregated miRNAs have been found in prostate cancer, such as miR-221/222, miR-143/145, miR-23b/27b/24-1 and miR-1/133a, which were down-regulated and had tumor inhibiting functions [15]. A previous study found that circulating miR-590-5p could be used as routine diagnostic tools for lung cancer, and as a potential prognostic marker for liquid biopsy. Besides, overexpression of miR-590-5p reduced the development of NSCLC cells, and regulated the expression of epithelial-mesenchymal transformation (EMT)-related proteins by targeting the signal transducers and activators of transcription 3 (STAT3) [16]. However, the precise mechanism by which miR-590-5p affects NSCLC needs further investigation.

Thrombospondin 2 (THBS2), as a secreted protein, was confirmed to be highly expressed in different cancers, including cervical cancer [17], colorectal cancer [18] and NSCLC [19]. A previous report suggested that THBS2 was involved in the proliferation, apoptosis and anti-autophagy regulation of cervical cancer cells by miR-20a [20]. Tian et al. found the expression and clinicopathological features of THBS2 in colorectal cancer were significantly correlated with the prognosis of cancer and might be used as a biomarker of prognosis [21]. However, the molecular function of THBS2 in NSCLC remains poorly defined.

In this study, the targeting relationship between circ_0020123 and miR-590-5p was firstly detected. The effects of circ_0020123 on cell proliferation, migration, apoptosis and tumor growth were performed by gain- and loss-of-function experiments and molecular biology techniques.

## Materials and methods

### Patients and specimens

NSCLC tissues and the adjacent healthy lung tissues were taken from 42 NSCLC patients in the Lianyungang Second People’s Hospital. All volunteers signed written informed consents. NSCLC tissues and the adjacent tissues were immediately frozen in liquid nitrogen and kept at − 80 °C for further experiments. This research was approved by the Ethics Committee of Lianyungang Second People’s Hospital.

### Cell culture and cell transfection

Two NSCLC cell lines (A549 and H1299) and one normal lung cell line (IMR90) were obtained from the Beijing Concorde Cell Library (Beijing, China). A549, H1299 and IMR90 cells were cultivated in Dulbecco’s modified eagle medium (DMEM) (HyClone, Logan, UT, USA) supplementing with 10% fetal bovine serum (FBS, HyClone) and cultured in an incubator at 37 ℃ with 5% CO_2_.

Small interfering RNA (siRNA) targeting circ_0020123-1 (si-circ_0020123-1 and si-circ_0020123-2), short hairpin RNA (shRNA) targeting circ_0020123 (sh-circ_0020123), miR-590-5p-inhibitors, siRNA negative control (si-NC), sh-NC and NC-inhibitors were all obtained from Biomics Biotech (Jiangsu, China). Full length of THBS2 cDNA (Sangon Biotech, Shanghai, China) was subcloned into pcDNA3.1 plasmid (EK-Bioscience, Shanghai, China). Then, cell transfection was performed by Lipofectamine 2000 (Thermo Fisher Scientific, Waltham, MA, USA).

### RNA isolation and quantitative real-time polymerase chain reaction (qRT-PCR)

The TRIzol reagent (Invitrogen, Carlsbad, CA, USA) was used for extracting the total RNAs. Next, the reversed transcription was carried out by RT-PCR kit (Invitrogen). The qRT-PCR was performed using the ABI SYBR Green Master Mix (Invitrogen). The primers in our study were as follows: F-5′-TTCGGACGACCGTCAAACAT-3′ and R-5′-AGGATCCCTGCACCACAATG-3′ for circ_0020123, F-5′-TGAAAGACGTGATGGCACAC-3′ and R-5′-CTTCCATTTTGGGGTTTTTGG-3′ for miR-590-5p, F-5′-AGAAGGCTGGGGCTCATTTG-3′, R-5′-AGGGGCCATCCACAGTCTTC-3′ for glyceraldehyde-3-phosphate dehydrogenase (GAPDH) [22], F-5′-GCGGCTGGGTCTATTTGTC-3′ and R-5′-GCAGGAGGTGAAGAACCATC-3′ for THBS2 [23]. F-5′-ATTGGAACGATACAGAGAAGATT-3′ and R-5′-GGAACGCTTCACGAATTTG-3′ for U6 [24]. GAPDH and U6 were the internal parameters.

### Cell Counting Kit-8 (CCK-8) assay

The proliferation viability of A549 and H1299 cells were detected by the Cell-Counting Kit-8 (MSK, Wuhan, China). A549 and H1299 cells were cultivated into a 96-well plate with a density of 5 × 10^4^ cells/well and incubated in 37 °C for 0, 24, 48 or 72 h. Then, 100 μL fresh medium and CCK-8 solution was added. After incubation at 37 °C for 4 h, the OD 490 values were detected by the Multiskan Ascent 354 microplate reader (Abcam, Cambridge, MA, USA).

### Transwell assay

Transwell chamber (Corning Life Sciences, Corning, NY, USA) was used to detect cell migration. Firstly, the serum-free DMEM (Thermo Fisher Scientific) was fixed with cell suspension (10,000 cells) and seeded into the upper chamber, and the DMEM containing 10% serum was put into the lower chamber. After incubation for 6 h, the cells in lower surface of the upper chamber were treated with 4% formaldehyde solution for 2.5 h, and then thoroughly washed. Finally, the migrated cells were stained with crystal violet (Corning Life Sciences) and observed by using a microscope.

### Flow cytometry

Firstly, A549 and H1299 cells were cultured and PBS was used for washing cells. Then, the binding buffer was used to resuspend cells, and the Annexin V-fluorescein isothiocyanate (V-FITC)/propidium iodide (PI) Apoptosis Detection Kit (Thermo Fisher Scientific) was used to stain cells. Finally, cell apoptosis was detected by flow cytometry (Thermo Fisher Scientific).

### Western blot analysis

The total proteins of NSCLC tumors or cells were collected by RIPA lysis buffer (Sangon Biotech). Then, the proteins were separated by 10% Sodium dodecyl sulphate polyacrylamide gel electrophoresis (SDS-PAGE) and transferred to polyvinylidene fluoride (PVDF) membranes (Thermo Fisher Scientific). The 5% skimmed milk was added and incubated with primary anti-GAPDH antibody (1:1000, Invitrogen, Carlsbad, CA, USA), anti-β-actin antibody (1:1000, Invitrogen), anti-Ki-67 antibody (1:1000, Invitrogen), anti-matrix metalloprotein-9 (MMP-9) antibody (1:1000, Invitrogen), anti-Cleaved-caspase9 (Cleaved-casp9) antibody (1:1000, Invitrogen) or anti-THBS2 antibody (1:1000, Invitrogen) at 4 °C overnight. Finally, the membranes were incubated with the secondary antibody for 1 h at room temperature. The results were viewed using Kodak film developer (Fujifilm, Japan).

### Dual-luciferase reporter assays

The wild type circ_0020123 sequences (circ_0020123-WT), mutant circ_0020123 sequences (circ_0020123-MUT), wild type THBS2 3′UTR sequences (THBS2-WT), mutant THBS2 3′UTR sequences (THBS2-MUT) were cloned into pGL-3 luciferase reporter plasmid (Promega, Madison, WI, USA). Then, the plasmid and miR-590-5p or miR-NC were co-transfected into A549 and H1299 cells by Lipofectamine 2000 (Thermo Fisher Scientific). After transfection for 36 h, the Dual-Luciferase Reporter Assay System (Promega) was performed to detect the luciferase activity.

### RNA immunoprecipitation (RIP)

Firstly, the Magna RIP RNA-binding Protein Immunoprecipitation Kit (gzscbio, Guangzhou, China) was performed to verify the relationship between circ_0020123 and miR-590-5p. In brief, the magnetic beads and anti-Ago2 antibody (Abcam) were added into cells and incubated for 24 h. Then, the proteinase K and the phenol–chloroform-isoamyl alcohol reagent were added for purifying RNAs. Finally, qRT-PCR was used to measure circ_0020123 enrichment.

### Animal experiments

The 4-week-old BALB/c male nude mice (Vitalriver, Beijing, China) were raised in a sterile environment for experiments. Then PBS was used to suspend A549 cells (2 × 10^6^) transfected with sh-circ_0020123 or sh-NC. Next, the nude mice were divided into two groups (n = 6). A549 cells transfected with sh-circ_0020123 or sh-NC were sh-circ_0020123 or sh-NC inoculated into the nude mice. The tumor volume was detected every 7 days. After 42 days, the nude mice were euthanatized and the tumor weight was detected. Besides, the tumor tissues from each group were collected to detect the expression of circ_0020123, miR-590-5p and THBS2. The animal experiment was approved by the Animal Experimentation Ethics Committee of Lianyungang Second People’s Hospital.

### Statistical analysis

The software GraphPad Prism 7 was performed for statistical analysis. The data was displayed as mean ± standard deviation (SD). The significant difference was calculated by Student’s *t* test and one-way analysis of variance (ANOVA). *P < 0.05 was considered as statistically significant.

## Results

### Circ_0020123 was upregulated in NSCLC tissues and cells

**Fig. 1:**
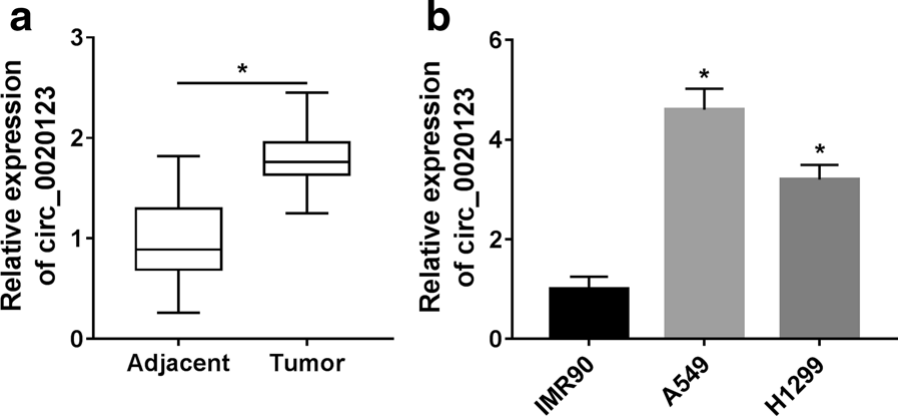
Circ_0020123 was upregulated in NSCLC tissues and cells. **a** QRT-PCR was used to detect the expression of circ_0020123 in adjacent healthy tissues (n = 42) and tumor tissues (n = 42). **b** The expression of circ_0020123 in normal cell line (IMR90) and NSCLC cell lines (A549 and H1299) was detected by qRT-PCR. *P < 0.05

To begin with, qRT-PCR was used to detect the expression of circ_0020123, the result showed that circ_0020123 was significantly upregulated in NSCLC tissues compared with the adjacent healthy tissues (Fig. 1a). Similarly, the expression of circ_0020123 in NSCLC cells (A549 and H1299) was markedly higher than that in normal cells (IMR90) (Fig. 1b). From these data, it is speculated that circ_0020123 might be acted as an oncogene in NSCLC.

### Downregulation of circ_0020123 inhibited the proliferation, migration and induced apoptosis of NSCLC cells

**Fig. 2:**
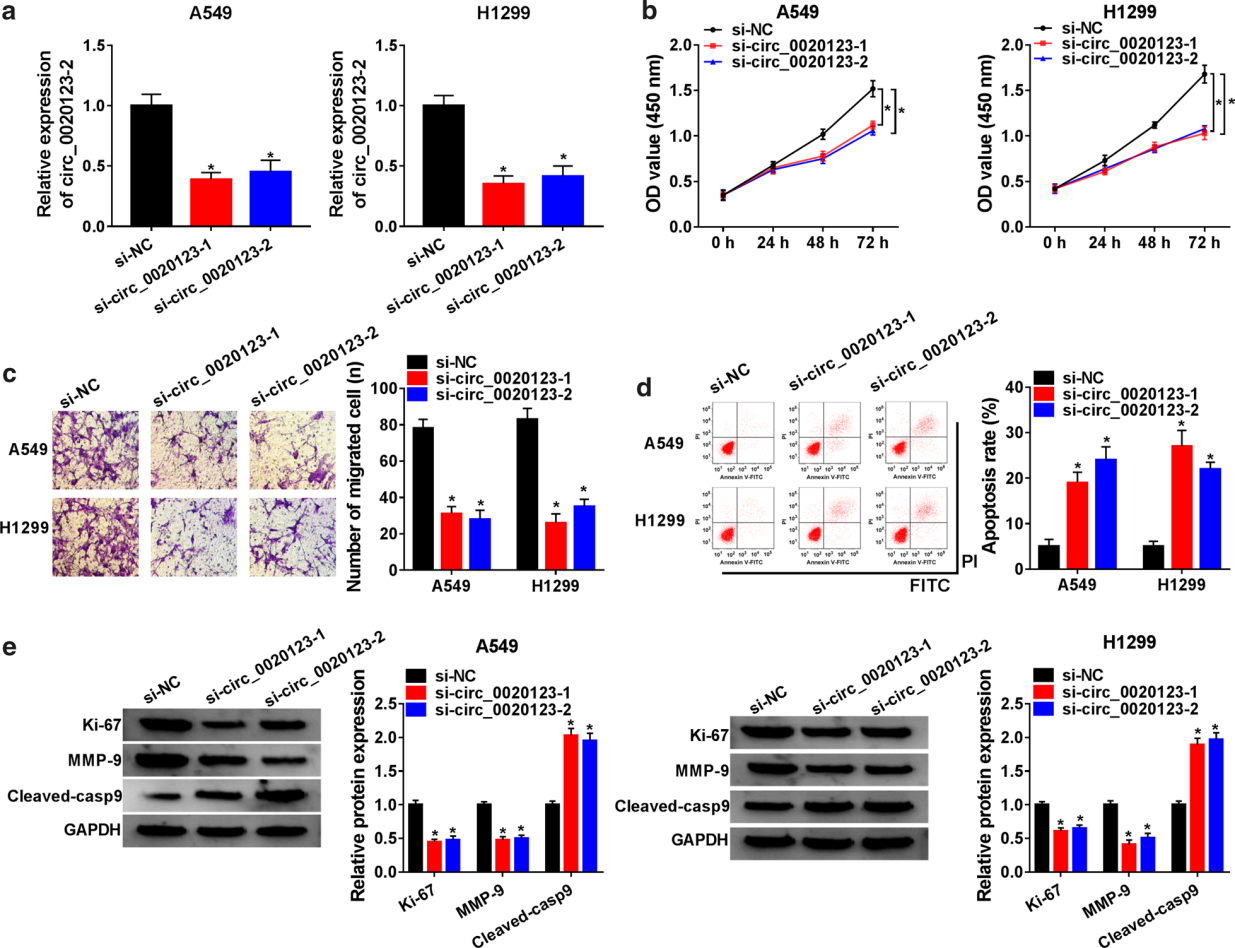
Downregulation of circ_0020123 inhibited the proliferation and migration and induced the apoptosis of NSCLC cells. **a** The transfection efficiency of si-circ_0020123-1 and si-circ_0020123-2 in A549 and H1299 cells was detected by qRT-PCR. **b** CCK-8 assay was used to detect the proliferation of A549 and H1299 cells transfected with si-circ_0020123-1 or si-circ_0020123-2. **c** The migration of A549 and H1299 cells transfected with si-circ_0020123-1 or si-circ_0020123-2 was measured by Transwell assay. **d** Flow cytolysis assay was used to detect the apoptosis of A549 and H1299 cells transfected with si-circ_0020123-1 or si-circ_0020123-2. **e** The protein levels of cell proliferation related protein Ki-67, cell migration related protein MMP-9 and cell apoptosis related protein Cleaved-casp9 were detected by Western blot. *P < 0.05

To investigate the functional effects of circ_0020123 on NSCLC cells, si-circ_0020123-1 and si-circ_0020123-2 were obtained and transfected into A549 and H1299 cells. Firstly, the transfection efficiency was detected by qRT-PCR (Fig. 2a). Next, CCK-8 was used to detect the proliferation, and the results showed that the proliferation of A549 and H1299 cells transfected with si-circ_0020123-1 or si-circ_0020123-2 was reduced (Fig. 2b). Moreover, the migration of A549 and H1299 cells was significantly downregulated by circ_0020123 knockdown (Fig. 2c). In addition, the apoptosis of A549 and H1299 cells transfected with si-circ_0020123-1 or si-circ_0020123-2 was obviously higher than transfected with si-NC (Fig. 2d). Finally, the protein levels of cell proliferation-related protein Ki-67 and cell migration-related protein MMP-9 were inhibited, while cell apoptosis-related protein Cleaved-casp9 was upregulated in NSCLC cells transfected with si-circ_0020123-1 or si-circ_0020123-2 (Fig. 2e). These data suggested that inhibition of circ_0020123 suppressed cell proliferation, migration and promoted apoptosis in NSCLC cells.

### Circ_0020123 directly targeted miR-590-5p

**Fig. 3:**
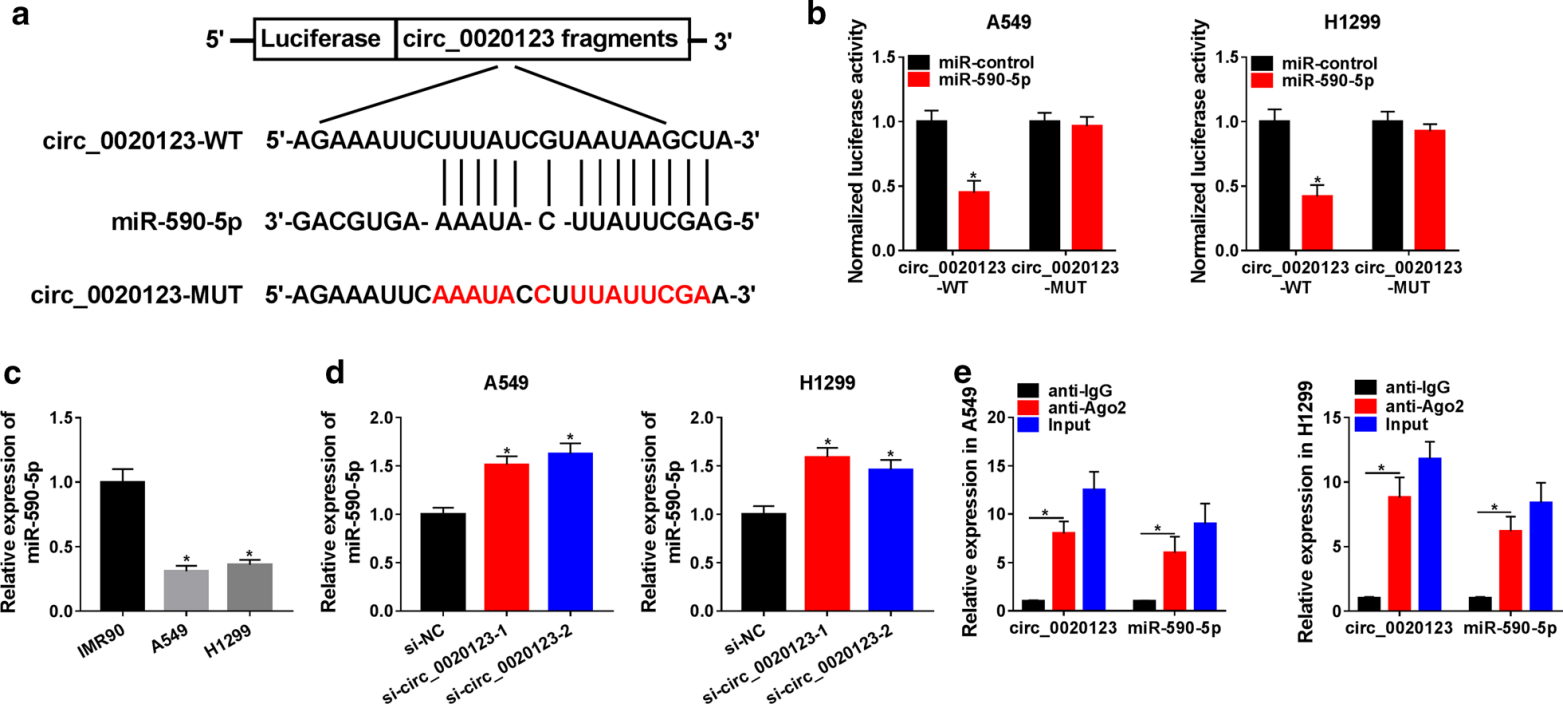
Circ_0020123 directly targeted miR-590-5p. **a** The binding site between circ_0020123 and miR-590-5p was detected by the online software starBase 3.0. **b** The luciferase activity of circ_0020123-WT or circ_0020123-MUT reporter plasmid in A549 and H1299 cells transfected with miR-NC or miR-590-5p was detected by dual-luciferase reporter assay. **c** QRT-PCR was used to detect the expression of miR-590-5p in A549 and H1299 cells. **d** The expression of miR-590-5p in A549 and H1299 cells transfected with si-circ_0020123-1 or si-circ_0020123-2 was detected by qRT-PCR. **e** RIP assay was used to confirm the relationship between circ_0020123 and miR-590-5p. *P < 0.05

By searching in the online software starBase 3.0, the potential binding sites between circ_0020123 and miR-590-5p were detected (Fig. 3a). To confirm that, the dual-luciferase reporter assay was performed, the results showed that the luciferase activity of circ_0020123-WT reporter plasmid was reduced by miR-590-5p mimic, while the circ_0020123-MUT reporter plasmid activity was not changed in A549 and H1299 cells (Fig. 3b). Furthermore, the expression of miR-590-5p was lower in A549 and H1299 cells compared with that in IMR90 cells (Fig. 3c). In contrast, miR-590-5p expression was elevated in A549 and H1299 cells transfected with si-circ_0020123-1 or si-circ_0020123-2 (Fig. 3d). Finally, the RIP assay was also used to confirm the targeting relationship between circ_0020123 and miR-590-5p, and the results showed that circ_0020123 and miR-590-5p were enriched in anti-Ago2 group (Fig. 3e).

### MiR-590-5p downregulation reversed the inhibition effects of circ_0020123 on NSCLC cells

**Fig. 4:**
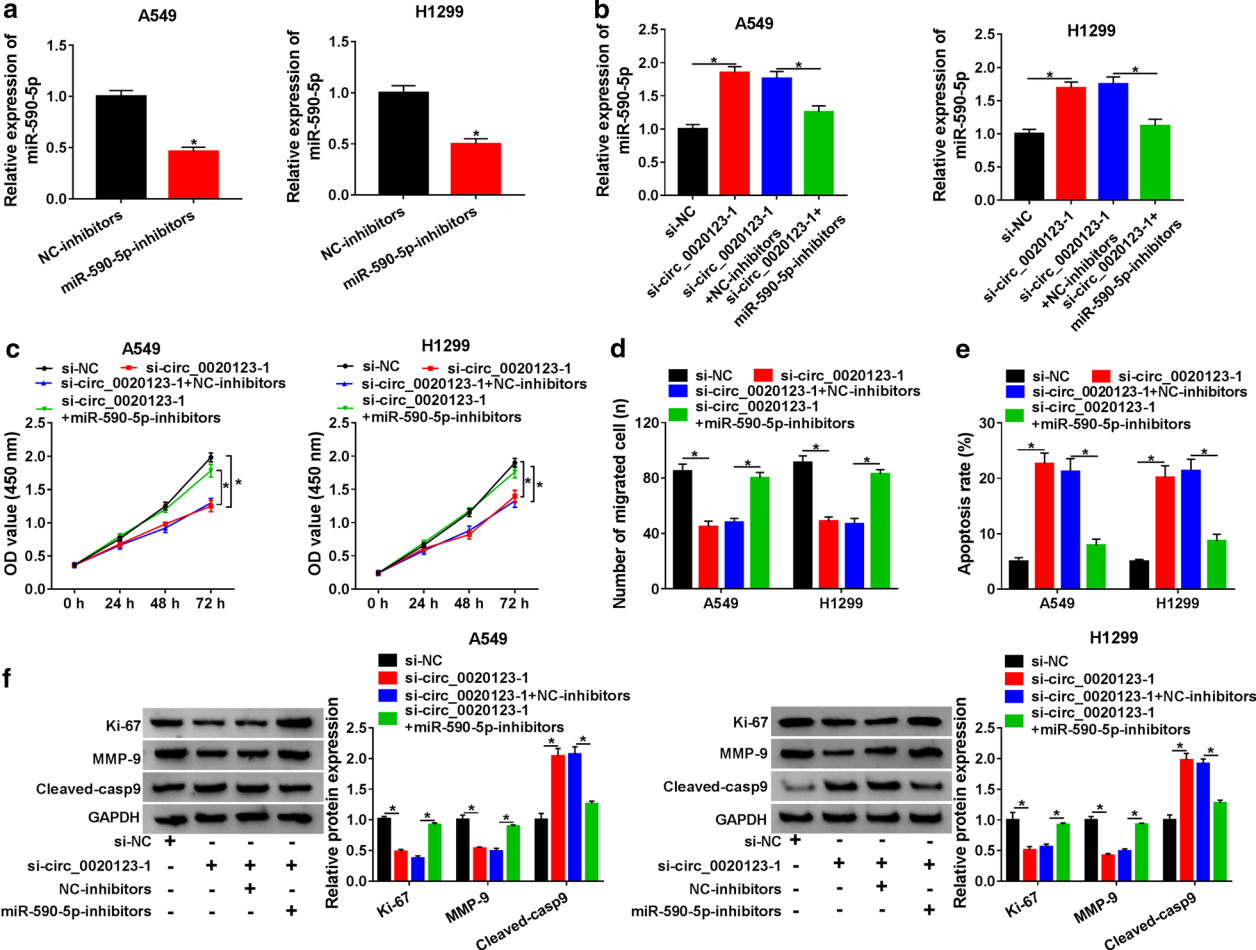
MiR-590-5p downregulation reversed circ_0020123 knockdown effects in NSCLC cells. **a** QRT-PCR was used to detect the expression of miR-590-5p in A549 and H1299 cells transfected with miR-590-5p-inhibitors. **b** The expression of miR-590-5p in A549 and H1299 cells transfected with si-circ_0020123-1 or si-circ_0020123-1 + miR-590-5p-inhibitors was detected by qRT-PCR. **c** The proliferation of A549 and H1299 cells transfected with si-circ_0020123-1 or si-circ_0020123-1 + miR-590-5p-inhibitors was tested by CCK-8 assay. **d** Transwell assay was used to measure the migration of A549 and H1299 cells transfected with si-circ_0020123-1 or si-circ_0020123-1 + miR-590-5p-inhibitors. **e** Flow cytolysis assay was used to detect the apoptosis of A549 and H1299 cells transfected with si-circ_0020123-1 or si-circ_0020123-1 + miR-590-5p-inhibitors. **f** The protein levels of Ki-67, MMP-9, Cleaved-casp9 in A549 and H1299 cells transfected with si-circ_0020123-1 or si-circ_0020123-1 + miR-590-5p-inhibitors were detected by Western blot. *P < 0.05

To further explore the functional effects between circ_0020123 and miR-590-5p, miR-590-5p-inhibitor was established. QRT-PCR was used to detect the transfection efficiency (Fig. 4a). Interestingly, miR-590-5p was upregulated in A549 and H1299 cells transfected with si-circ_0020123-1, while the expression of miR-590-5p was recovered in cells transfected with si-circ_0020123-1 + miR-590-5p-inhibitors (Fig. 4b). Moreover, circ_0020123-1 knockdown inhibited cell proliferation and migration, while the miR-590-5p inhibitor reversed these effects (Fig. 4c, d). In addition, the apoptosis of A549 and H1299 cells transfected with si-circ_0020123-1 was increased, which was abolished by miR-590-5p-inhibitor (Fig. 4e). Similarly, miR-590-5p inhibitors reversed the effects on the protein levels of Ki-67, MMP-9 and Cleaved-casp9 in A549 and H1299 cells transfected with si-circ_0020123-1 (Fig. 4f). These results indicated that miR-590-5p downregulation reversed the effects of circ_0020123 downregulation on the proliferation, migration and apoptosis of NSCLC cells.

### MiR-590-5p targeted THBS2 in NSCLC cells

**Fig. 5:**
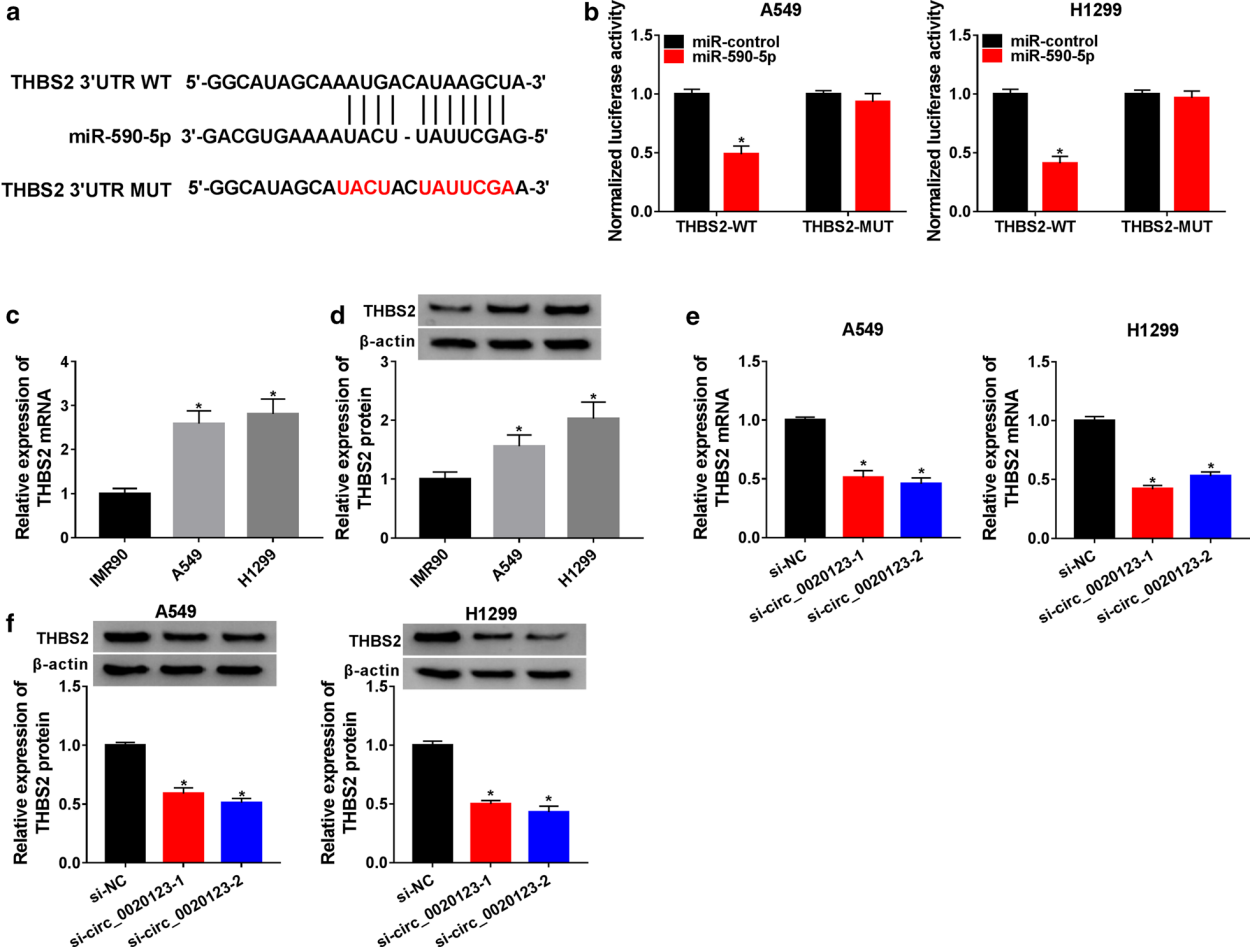
MiR-590-5p targeted THBS2 in NSCLC cells. **a** The potential binding site between THBS2 3′UTR and miR-590-5p was predicted by the online software TargetScan. **b** Dual-luciferase reporter assay was used to measure the luciferase activity of THBS2-WT or THBS2-MUT reporter plasmid in A549 and H1299 cells transfected with miR-NC or miR-590-5p. **c** QRT-PCR was used to detect the mRNA expression of THBS2 in NSCLC cells. **d** The protein level of THBS2 in NSCLC cells was tested by Western blot. **e** The mRNA expression of THBS2 in A549 and H1299 cells transfected with si-circ_0020123-1 or si-circ_0020123-2 was detected by qRT-PCR. **f** Western blot was used to measure the protein level of THBS2 in A549 and H1299 cells transfected with si-circ_0020123-1 or si-circ_0020123-2. *P < 0.05

The THBS2 3′UTR was predicted to contain the binding sites of miR-590-5p through the online software TargetScan (Fig. 5a). Then, the dual-luciferase reporter assay was used to confirm the targeting relationship. The results showed that co-transfection of miR-590-5p and THBS2-WT significantly limited the luciferase activity in both A549 and H1299 cells, the luciferase activity was not altered in cells co-transfected with miR-590-5p and THBS2-MUT (Fig. 5b). Importantly, the mRNA and protein level of THBS2 was enahnced in NSCLC cells (Fig. 5c, d). We further explored whether circ_0020123 affected the functions of THBS2 in NSCLC cells. The mRNA and protein expression of THBS2 were repressed in A549 and H1299 cells transfected with si-circ_0020123-1 or si-circ_0020123-2 (Fig. 5e, f).

### THBS2 overexpression reversed the effects of circ_0020123 knockdown on the proliferation, migration and apoptosis of NSCLC cells

**Fig. 6:**
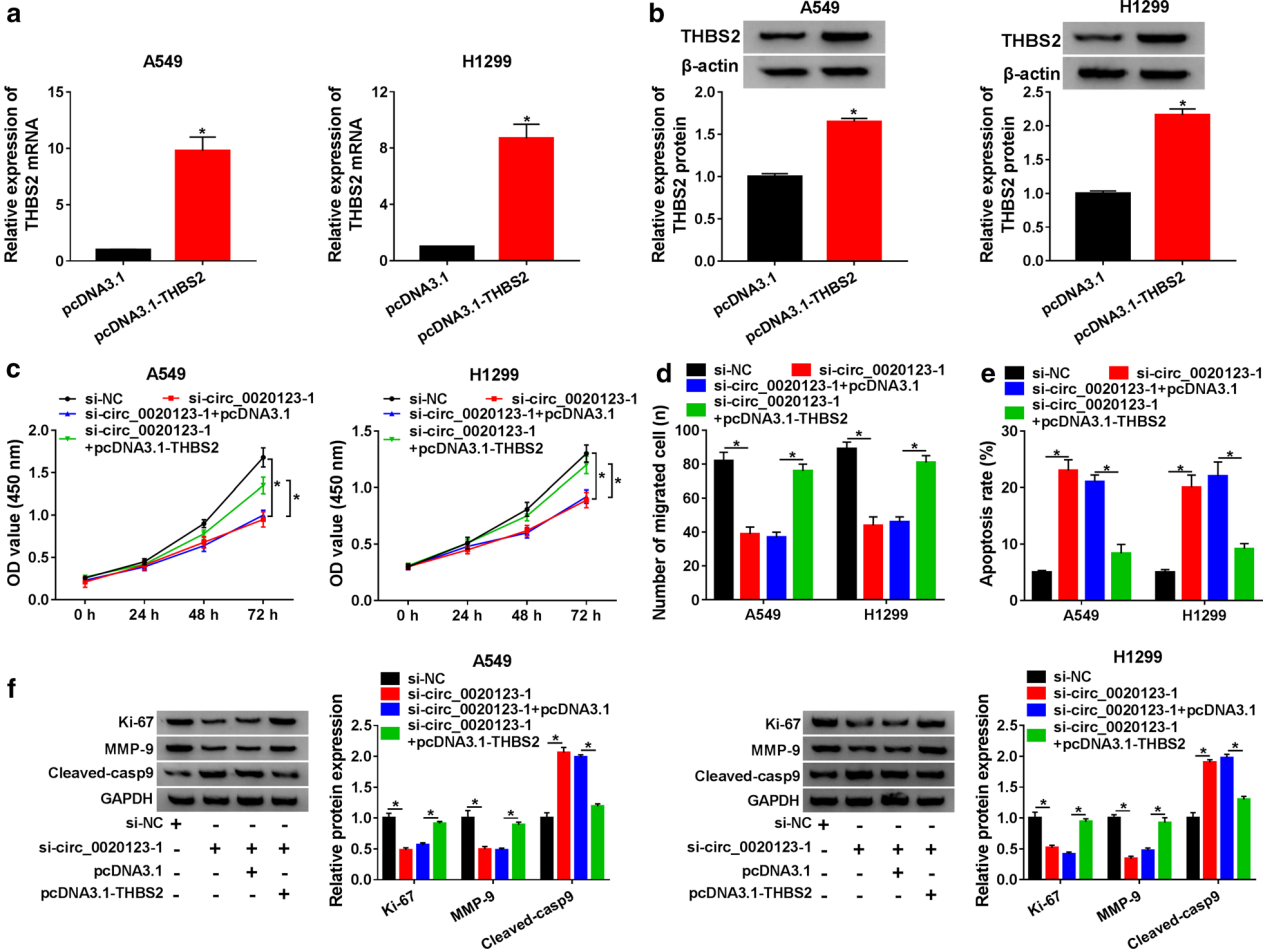
Overexpression of THBS2 reversed the effects of circ_0020123 knockdown on proliferation, migration and apoptosis of NSCLC cells. **a**, **b** The mRNA and protein expression of THBS2 in A549 and H1299 cells transfected with pcDNA3.1-THBS2 was detected by qRT-PCR and Western blot. **c** CCK-8 assay indicated the proliferation of A549 and H1299 cells transfected with si-circ_0020123-1 or si-circ_0020123-1 + pcDNA3.1-THBS2. **d** The migration of A549 and H1299 cells transfected with si-circ_0020123-1 or si-circ_0020123-1 + pcDNA3.1-THBS2 was measured by Transwell assay. **e** The apoptosis of A549 and H1299 cells transfected with si-circ_0020123-1 or si-circ_0020123-1 + pcDNA3.1-THBS2 was detected by Flow cytolysis assay. **f** The protein levels of Ki-67, MMP-9, Cleaved-casp9 in A549 and H1299 cells transfected with si-circ_0020123-1 or si-circ_0020123-1 + pcDNA3.1-THBS2 were detected by Western blot. *P < 0.05

Based on the work ahead of us, the pcDNA3.1-THBS2 was constructed. Then, the qRT-PCR and Western blot were used to detect the transfection efficiency, and the THBS2 expression was increased in A549 and H1299 cells transfected with pcDNA3.1-THBS2 (Fig. 6a, b). In addition, the proliferation and migration rates of A549 and H1299 cells transfected with si-circ_0020123-1 + pcDNA3.1-THBS2 were higher than that transfected with si-circ_0020123-1 (Fig. 6c, d). Meanwhile, a similarly phenomenon was also observed in cell apoptosis, the pcDNA3.1-THBS2 significantly reversed the promotion effect of circ_0020123 deletion on cell apoptosis (Fig. 6e). Furthermore, the effects of circ_0020123 deletion on Ki-67, MMP-9 and Cleaved-casp9 protein levels were also reversed by THBS2 overexpression (Fig. 6f). These data suggested that overexpression of THBS2 could reverse the effects of circ_0020123 downregulation on cell proliferation, migration and apoptosis.

### Reduction of circ_0020123 suppressed tumor growth in vivo through circ_0020123/miR-590-5p/THBS2 axis

**Fig. 7:**
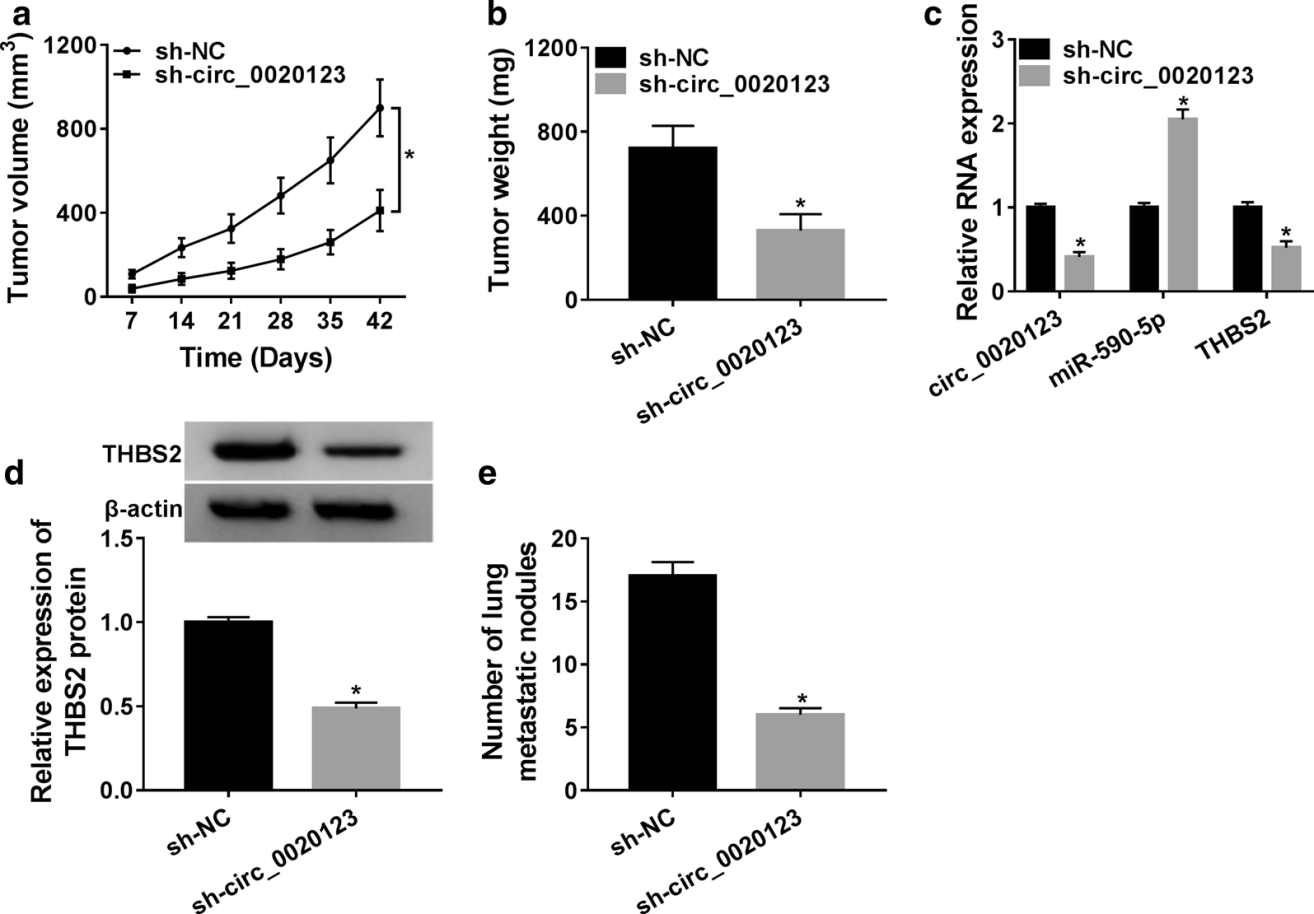
Reduction of circ_0020123 suppressed the tumor growth in vivo through circ_0020123/miR-590-5p/THBS2 axis. **a** A total of 2 × 10^6^ A549 cells transfected with sh-circ_0020123 or sh-NC were injected into nude mice to establish the xenograft tumor. Tumor volume was measured every 7 d after injection. **b** Tumor weight was measured on d 42. **c** The expression of circ_0020123, miR-590-5p and THBS2 in xenograft tumor was measured by qRT-PCR. **d** The protein level of THBS2 in xenograft tumor was evaluated by Western blot. **e** The number of lung metastatic nodules in xenograft tumor was detected by digital tomosynthesis (DTS). *P < 0.05

To further explore the function of circ_0020123 in NSCLC cells, the sh-circ_0020123 was constructed and the xenograft tumor was established. Then, A549 cells transfected with sh-circ_0020123 or sh-NC were injected into the nude mice. The xenograft tumor volume was measured every 7 days after injection, and the results showed that tumor volume was smaller sh-circ_0020123 group than that in sh-NC group (Fig. 7a). Moreover, tumor weight was inhibited by circ_0020123 knockdown (Fig. 7b). Furthermore, the expression circ_0020123 and THBS2 was decreased, while the miR-590-5p was increased in xenograft tumor transfected with sh-circ_0020123 (Fig. 7c). Western blot assay also revealed that the protein level of THBS2 was repressed by circ_0020123 knockdown (Fig. 7d). Finally, the digital tomosynthesis (DTS) was used to detect the number of lung metastatic nodules in xenograft tumor, and it was reduced in sh-circ_0020123 group (Fig. 7e). The results suggested that downregulation of circ_0020123 inhibited tumor growth in vivo.

## Discussion

Clinically, only a few NSCLC patients were diagnosed at an early stage and treated by surgical resection. More than 60% of NSCLC patients were diagnosed with the advanced stage or metastatic tumors [25]. Thus, finding novel biomarkers and therapeutic targets were necessary for the effective diagnosis and treatment of NSCLC.

Recently, circRNA was no longer considered as a random product in the RNA shearing process, and its biological significance and function in malignant tumors had received more and more attention. Previous reports revealed that circ_0020123 was involved in the development of NSCLC [26]. Moreover, the level of circ_0020123 was elevated in NSCLC cells [13]. Consistently, we found that the expression of circ_0020123 was markedly higher in NSCLC tissues and cells. Moreover, this research indicated that inhibition of circ_0020123 suppressed the proliferation, migration and induced apoptosis of NSCLC cells in vitro. Besides, circ_0020123 promoted tumor growth in vivo.

Endogenous circRNAs could act as microRNA sponges to inhibit their function, and some studies linked miRNA sponges to human diseases, including cancer [27]. A previous study indicated that circRNA c-transferrin receptor (cTFRC) regulated TFRC by sponging miR-107 to facilitate bladder carcinoma development [28]. MiR-590-5p was studied in different cells, such as airway smooth muscle cells [22], colon epithelial cells [29] and NSCLC cells [30]. However, the potential relationship between miR-590-5p and circRNA has not been researched. In this study, circ_0020123 directly targeted miR-590-5p and miR-590-5p inhibition reversed the effects of circ_0020123 knockdown on NSCLC progression. These data provided a clue to the therapeutic strategy for NSCLC.

Our study also confirmed that miR-590-5p could target THBS2 directly in NSCLC cells. THBS2 is a calcium-binding protein that binds to and inactivates matrix metalloproteinase (MMP) genes involved in tissue formation and repair [31, 32]. A previous document suggested that THBS2 acted as a target of miR-221-3p and participated in lymph node metastasis in cervical cancer [33]. The data in this research showed that the expression of THBS2 in NSCLC cells was markedly higher than normal healthy cells. Furthermore, overexpression of THBS2 reversed the effects of circ_0020123 knockdown on proliferation, migration and apoptosis of NSCLC cells, suggesting that circ_0020123 promoted the progression of NSCLC cells through miR-590-5p/THBS2 axis.

## Conclusion

In conclusion, our research showed that the expression of circ_0020123 was higher in NSCLC tissues and cells than control, and downregulation of circ_0020123 inhibited the proliferation, migration and promoted apoptosis of NSCLC cells, and also suppressed tumor growth in vivo. Moreover, circ_0020123 directly targeted miR-590-5p, while miR-590-5p inhibition reversed the effects of circ_0020123 knockdown on NSCLC cells. More importantly, circ_0020123 regulated the expression of THBS2 by sponging miR-590-5p, and upregulation of THBS2 reversed the effects of circ_0020123 knockdown on NSCLC cells. Therefore, our research demonstrated that circ_0020123 enhanced proliferation, migration and inhibited apoptosis of NSCLC cells by sponging miR-590-5p to regulate THBS2.

## Data Availability

Please contact corresponding author for data requests.

## Abbreviations

NSCLC: Non-small cell lung cancer
circRNA: Circular RNA
qRT-PCR: Quantitative real-time polymerase chain reaction
CCK-8: Cell Counting Kit-8
MMP-9: Matrix metalloprotein-9 Cleaved-casp9: Cleaved-caspase9
Cleaved-casp9: Cleaved-caspase9
RIP: RNA immunoprecipitation
ZEB1: Zinc-finger-enhancer binding protein 1
EZH2: Zeste homolog 2
STAT3: Signal transducers and activators of transcription 3
THBS2: Thrombospondin 2
